# The efficacy of traditional Chinese medicine on immune function in patients with hepatitis B-related liver fibrosis or cirrhosis: a systematic review and meta-analysis

**DOI:** 10.3389/fmed.2026.1872781

**Published:** 2026-07-20

**Authors:** Zhulin Wu, Wanjun Tan, Beier Luo, Xiaowei Liu, Weiqing Zhang, Lianan Wang, Mingbo Lai

**Affiliations:** 1 Department of Traditional Chinese Medicine, People's Hospital of Longhua, Shenzhen, China; 2 The Eighth Affiliated Hospital, Sun Yat-Sen University, Shenzhen, China; 3 Bethune Third Clinical Medical College, Jilin University, Changchun, China; 4 Department of Traditional Chinese Medicine, The University of Hong Kong-Shenzhen Hospital, Shenzhen, China

**Keywords:** Chinese medicine, hepatic fibrosis, hepatitis B, immune, liver cirrhosis, randomized controlled trials

## Abstract

**Objective:**

Currently, there is no effective treatment to reverse hepatitis B-related liver fibrosis or cirrhosis. Current nucleos(t)ide analogs (NAs) control but rarely eliminate hepatitis B virus (HBV), leaving chronic hepatitis B at risk of developing liver fibrosis/cirrhosis. Traditional Chinese medicine (TCM) shows promise in slowing the progression of hepatitis B-related liver fibrosis/cirrhosis, but its impact on immune function remains debated.

**Methods:**

The electronic databases, including Chinese National Knowledge Infrastructure, Wanfang, SinoMed, Weipu, PubMed, Web of Science, EMBASE, and Cochrane databases, were retrieved (April 7, 2026), and the randomized controlled trials (RCTs) that met the inclusion criteria were included. Methodologic quality assessment of the included RCTs was done based on the Cochrane RoB 2 tool. Subsequently, the valid data were screened and analyzed by meta-analysis with Review Manager 5.4.1, and the quality of the evidence was assessed using the GRADE method. This study was registered in the PROSPERO (CRD420261351373).

**Results:**

Finally, 25 RCTs were included, containing 2,585 patients with hepatitis B-related liver fibrosis or cirrhosis. Risk of bias evaluations suggested some concerns for the majority of RCTs. The results of meta-analysis indicated that TCM with NAs could improve CD3 + [MD = 5.83, 95%CI (3.93, 7.73), *p* < 0.00001], CD4 + [MD = 4.32, 95%CI (3.56, 5.08), *p* < 0.00001], CD4+/CD8 + [MD = 0.24, 95%CI (0.18, 0.29), *p* < 0.00001], and NK cells, and decrease CD8 + cells [MD = −2.67, 95%CI (−3.46, −1.89), *p* < 0.00001], interleukin (IL)-6, transforming growth factor (TGF-*β*), tumor necrosis factor-*α* (TNF-α), hyaluronic acid (HA), laminin (LN), type IV collagen (IV-C), type III procollagen (PC-III), liver stiffness measurement (LSM), alanine aminotransferase (ALT), aspartate aminotransferase (AST), total bilirubin, and albumin in serum compared with NAs alone. Moreover, descriptive data showed that TCM was safe, and the funnel plot suggested the included research might have slight publication bias. The GRADE classification showed that the certainty of evidence was low for the immune function, liver function, and liver fibrosis indexes.

**Conclusion:**

A combination of NAs and TCM could improve immune cell metrics, cytokines, liver function indexes, and liver fibrosis indexes of patients with hepatitis B-related liver fibrosis or cirrhosis. Due to the low quality of research, more high-quality RCTs are needed to improve the level of evidence.

**Systematic review registration:**

https://www.crd.york.ac.uk/PROSPERO/view/CRD420261351373.

## 1 Introduction

Cirrhosis is a severe global health issue that can lead to liver failure and liver cancer. Cirrhosis has been steadily increasing over the last few decades (1, 2). Liver fibrosis, an early stage of cirrhosis, involves the activation of hepatic stellate cells (HSCs) and extracellular matrix buildup (3). As fibrosis advances, pseudolobules form, leading to cirrhosis. Hepatitis B virus (HBV) is an important cause of liver fibrosis and cirrhosis. HBV is a chronic infection that affects 250 million people globally, 10 to 15% of whom develop liver fibrosis and cirrhosis (4).

At present, the efficacy of treatments for hepatitis B-related liver fibrosis and cirrhosis remains constrained. While liver transplantation constitutes an effective intervention for advanced cirrhosis, its utilization is restricted by the scarcity of donor organs (5). Furthermore, despite recent advancements in the research of anti-fibrotic drugs, no specific anti-fibrotic agents have been approved for clinical application (6). At present, there is no gold standard for the treatment of liver fibrosis, and current therapeutic strategies primarily aim to manage the underlying cause, such as hepatitis B, and to prevent associated complications.

Although antiviral therapy can substantially reduce HBV DNA levels, its capacity to reverse liver fibrosis or cirrhosis remains limited. For instance, a study demonstrated that low-level viremia persisted in patients undergoing entecavir treatment, which correlated with the progression of liver fibrosis (7). This indicates that viral persistence, even under antiviral therapy, may contribute to unfavorable long-term liver outcomes. Moreover, recent research has demonstrated that the restructured immune landscape may facilitate disease progression in HBV-related liver fibrosis. HBV cirrhosis is accompanied by a concurrent reduction in cytotoxic natural killer (NK) cells and alterations in T cell subsets, such as an increase in CD8 + T cells and a decrease in CD4 + T cells (8). An in-depth investigation of these mechanisms could offer novel insights for clinical interventions aimed at regulating immune responses in treating hepatitis B-related liver fibrosis/cirrhosis. Future research should prioritize the integration of additional therapeutic modalities alongside antiviral treatment to more effectively enhance the prognosis of liver fibrosis and cirrhosis.

Traditional Chinese medicine (TCM), with a history of over 2000 years, is integral to China’s healthcare system and shows promise in treating hepatitis B-related liver fibrosis and cirrhosis. For instance, Fuzheng Huayu formula, a traditional Chinese patent medicine, is widely used clinically for liver fibrosis and cirrhosis caused by HBV, with the effects of improving serum liver function, liver pathological histology related to liver fibrosis (9). Chinese herbal formula Ruangan granule (RG) with entecavir can improve advanced liver fibrosis/early cirrhosis regression in patients with hepatitis B, further reducing the risk of hepatocellular carcinoma (10). However, evidence on TCM’s impact on immune function in these conditions remains limited and controversial.

Several randomized clinical trials (RCTs) have demonstrated that nucleos(t)ide analogs (NAs) combined with TCM could decrease CD8 + cells in treating hepatitis B-related liver fibrosis/cirrhosis (11, 12). However, another study showed NAs combined with TCM increase CD8 + cells in treating hepatitis B-related cirrhosis (13). Thus, we completed a systematic review to explore the efficacy of NAs combined with TCM in comparison with NAs alone for hepatitis B-related liver fibrosis/cirrhosis, in order to provide a scientific basis for the application of TCM.

## 2 Methods

### 2.1 Inclusion criteria

#### 2.1.1 Type of articles

The analysis was limited to RCTs, with or without blinding. This study was registered in PROSPERO, having Registration number CRD420261351373. No ethical statement will be required for this meta-analysis.

#### 2.1.2 Participants

All adults who met the diagnostic criteria of hepatitis B-related liver fibrosis/cirrhosis were enrolled. The following diagnostic methods and criteria were used: the “Guidelines for the Prevention and Treatment of Chronic Hepatitis B” [version 2015 (14), 2019 (15), or 2022 (16)] of China and “Guidelines for Diagnosis and Treatment of cirrhosis” (17) of China.

#### 2.1.3 Interventions

The control groups received treatment with NAs alone, and placebos of traditional Chinese medicine can be used. Additional treatments were administered in accordance with established hepatitis B guidelines. The experimental groups were administered oral TCM in conjunction with NAs. There were no restrictions on the duration or dosage of the treatments.

#### 2.1.4 Observation indicators

The observation indicators were as follows: (1) Included RCTs were required to report on immune cell metrics, including CD3+, CD4+, CD8+, CD4+/CD8 + ratios, NK cells, or cytokines such as interleukin (IL)-6, IL-8, transforming growth factor (TGF)-*β*, and tumor necrosis factor-*α* (TNF-α), as outcome measures; (2) indexes of liver fibrosis, including hyaluronic acid (HA), laminin (LN), type IV collagen (IV-C), type III procollagen (PC-III), and liver stiffness measurement (LSM); (3) liver function parameters, such as alanine aminotransferase (ALT), aspartate aminotransferase (AST), total bilirubin (TBIL), and albumin (ALB); (4) additional observation indicator: the virological marker (HBV-DNA); (5) safety was assessed descriptively through the evaluation of adverse events. According to the protocol registered on PROSPERO, the primary outcomes were defined as immune cells (CD3+, CD4+, CD8+, and CD4+/CD8+, NK) and cytokines (IL-2, IL-10, and interferon *γ* (IFN-γ)), and secondary outcomes comprised liver fibrosis indexes. Throughout the study, we found that data on IL-2, IL-10, and IFN-γ were not reported in the included studies, while data on other cytokines such as IL-6, IL-8, TGF-*β*, and TNF-*α* as alternatives were reported. Additionally, liver function and viral parameters were also included as secondary observations due to their clinical relevance.

### 2.2 Exclusion criteria

The exclusion criteria were defined as follows: (1) Patients with hepatitis B who have not yet developed liver fibrosis or cirrhosis; (2) patients diagnosed with liver cancer; (3) patients with other forms of viral hepatitis, such as hepatitis C; (4) women who are pregnant or breastfeeding; (5) intervention measures included other TCM treatments, such as auricular acupressure or acupuncture; (6) control groups received TCM therapy; (7) duplicate articles, plagiarized literature, or studies with incomplete data; (8) retrospective studies, case reports, theoretical studies, reviews, and experimental articles; (9) articles not published in Chinese or English.

### 2.3 Search strategy

A comprehensive search was conducted using English-language databases, including PubMed, Cochrane Library, Embase, and Web of Science, as well as Chinese-language databases, such as the China National Knowledge Infrastructure (CNKI), Wanfang Data, Weipu Database, and China Biomedical Literature (CBM) Database. These databases were searched for relevant articles from their inception until April 7, 2026. The search terms employed were “HBV OR hepatitis b,” “cirrhosis OR cirrhotic OR hepatocirrhosis OR hepatic fibrosis OR liver fibrosis,” “Chinese medicine OR Chinese materia medica,” “immune,” and “randomized,” with equivalent terms used for searches in Chinese databases. Detailed information on the search process is provided in Supplementary file S1.

### 2.4 Literature screening and quality evaluation

To minimize bias, the literature retrieved during the database search was independently selected by two authors (Zhulin Wu and Xiaowei Liu). Articles unrelated were excluded after reviewing their titles and abstracts. Subsequently, the quality of the remaining articles was assessed, and the extracted study data were cross-verified. The methodological quality of the RCTs was evaluated using the Cochrane Risk of Bias tool version 2 (Cochrane RoB 2 tool) (18), which encompasses five domains: (1) randomization process, (2) deviations from intended interventions, (3) missing outcome data, (4) measurement of the outcome, and (5) selection of the reported result. Study quality was categorized as “high risk,” “some concerns,” or “low risk.” Articles meeting all criteria were classified as having a low risk of bias, while those failing to meet any criteria were classified as having a high risk of bias. Articles that did not fully meet all criteria were classified as having some concerns regarding bias.

### 2.5 Data extraction

Data will be extracted independently by at least two authors with a process to resolve differences. A data extraction table was developed to systematically collect information from the literature, including authors, year, number of cases, intervention measures, treatment course, observation indicators, and adverse reactions of intervention measures. The data were organized using Excel 2016 software (Microsoft Office).

### 2.6 Statistical analyses

The Modern Medical Records Cloud Platform V3.0 (The Institute of Information on Traditional Chinese Medicine of the China Academy of Chinese Medical Sciences)^1^ was used to analyze the compositions (herbs) of TCM in the 25 RCTs. Statistical analysis and association rule mining were utilized to analyze the distribution characteristics of the herbs and discover the implicit association between them.

A meta-analysis was conducted to synthesize the findings of the included studies using Review Manager version 5.4.1. For dichotomous variables, the Risk Ratio was employed as the measure of effect size, while for continuous variables, the mean difference (MD) was utilized. The results were computed with 95% confidence intervals (95% CIs). Additionally, heterogeneity among the studies was evaluated using the I^2^ statistic. A fixed-effects model was applied when the I^2^ value was below 50%, whereas a random-effects model or descriptive analysis was used when the I^2^ value exceeded 50%. The meta-analysis results were presented through a forest plot, with *p*-values less than 0.05 considered statistically significant, and funnel plots were generated to assess publication bias. The GRADEproGDT online tool^2^ was utilized to evaluate the evidence quality for the outcome according to the five downgrading factors: risk of bias, inconsistency, indirectness, imprecision, and potential publication bias.

## 3 Results

### 3.1 Search results

**Figure 1:**
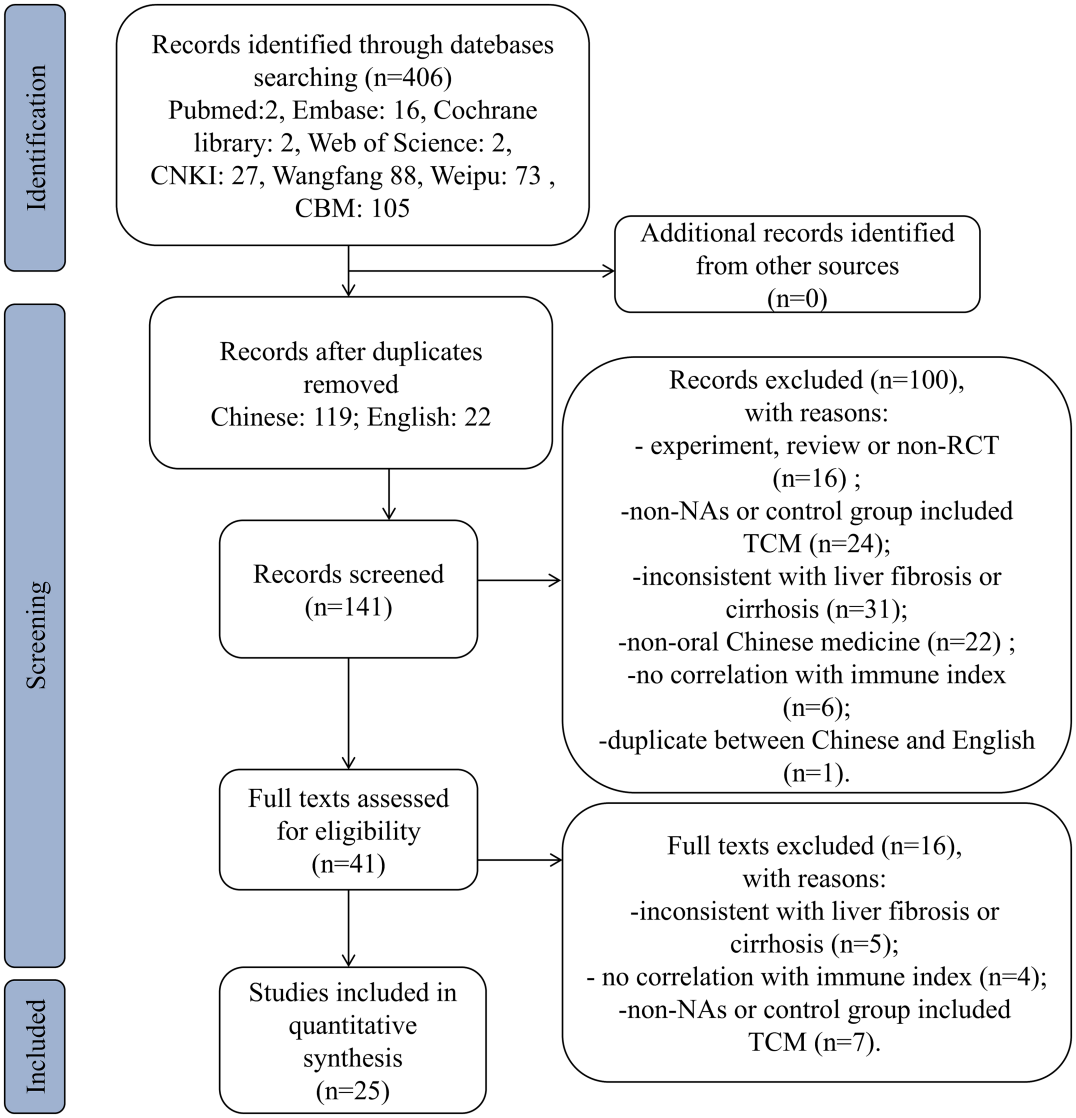
Flow chart of the study selection process.

Through the search of the Chinese databases, a total of 1,086 related articles were collected, comprising 27 from CNKI, 88 from Wanfang, 73 from Weipu, and 105 from CBM. Searches of English databases identified 2 articles from PubMed, 2 from Web of Science, 16 from EMBASE, and 2 from the Cochrane Library. Based on the established inclusion and exclusion criteria, 25 Chinese RCTs (11–13, 19–40) were selected for analysis (Figure 1).

### 3.2 The basic characteristics of the 25 RCTs

**Figure 2:**
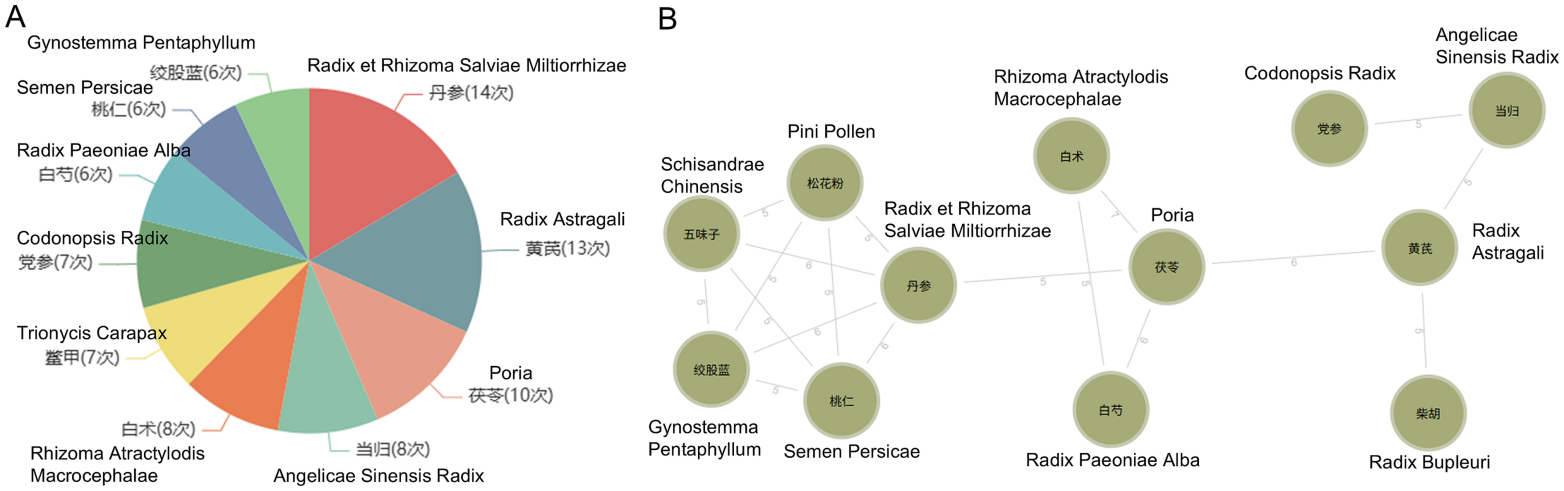
Frequency of herbs and association rule of herbs in the 25 RCTs. **(A)** The top 10 herbs were presented. **(B)** Visualization of significant association rules between herbs with minimum support (0.2) and confidence (0.5).

In this study, a total of 25 randomized controlled trials (RCTs) were selected, including 2,585 patients diagnosed with hepatitis B-related liver fibrosis or cirrhosis (Table 1). This meta-analysis investigated four observation indexes: immune index, liver fibrosis index, liver function, and other indexes. In the experimental group, patients received oral TCM in conjunction with NAs, whereas the control group was treated with NAs alone. Importantly, all studies included in the meta-analysis demonstrated comparable baseline characteristics (such as gender, age, and observation indicators) between the intervention and control groups. Moreover, compositions (herbs) of TCM in the 25 RCTs are shown in Table 2. Additionally, the top 10 herbs and visualization of significant association rules with minimum support (0.2) and confidence (0.5) were presented in Figure 2.

**Table 1:** The basic characteristics of the RCTs included.

Study	Sample (T/C)	Diagnosis	Intervention (T/C); course	Indicators	AR
Chen 2019 (19)	64/63	liver fibrosis	Fuzheng Huayu capsule+ETV/ETV; 48w	①②③	NA
Chen 2021 (20)	56/56	cirrhosis	Zishen Rougan decoction +ADV/ADV; 24w	①②③④	√
Duan 2021 (11)	45/45	liver fibrosis	Rougan Huaxian granules+ETV/ETV; 12w	①②③④	√
Feng 2022 (21)	47/46	cirrhosis	Fufang Biejia Ruangan tablet+ETV/ETV; 6 m	①②③	NA
Gao 2023 (22)	58/60	liver fibrosis	Yigan Huashi decoction +ETV/ETV; 24w	①②③	NA
Gong 2025 (23)	111/110	liver fibrosis	Anluo Huaxian pill+TAF/TAF; 6 m	①②③	NA
Guo 2025 (24)	41/41	cirrhosis	Tongluo Ruangan decoction +ETV/ETV; 3 m	①②	√
Jiang 2021 (25)	45/45	liver fibrosis	Chaishao Liujun decoction+ETV/ETV; 24w	①②③	NA
Li 2017 (26)	42/42	cirrhosis	TCM + ETV/ETV; 12 m	①②③	√
Li 2024 (27)	60/60	cirrhosis	Xiangsha Liujunzi decoction+ETV/ETV; 6 m	①②③	√
Lin 2025 (28)	40/40	cirrhosis	Fuzheng Huayu capsule+ETV/ETV; 6 m	①②③	√
Liu 2023 (29)	60/60	cirrhosis	Ruangan Kangxian Formula+ETV/ETV; 24w	①②③	√
Ma 2021 (30)	46/45	liver fibrosis	Rougan Huazhuo Bushen Formula+ADV/ADV; 6 m	①②③	NA
Niu 2023 (31)	64/64	cirrhosis	Fuzheng Huayu capsule +TDF/TDF; 48w	①②③	√
Song 2021 (32)	35/37	cirrhosis	Fuzheng Huayu capsule+ETV/ETV; 48w	①②③	NA
Sun 2018 (12)	43/43	cirrhosis	Yinzhi Ganfu granules+ ETV/ETV; 48w	①②③	√
Sun 2022 (33)	109/109	cirrhosis	Fuzheng Qudu Decoction +ETV/ETV; 12w	①③④	NA
Xie 2025 (34)	51/51	cirrhosis	Fuzheng Huayu capsule+ETV/ETV; 24w	①②③	NA
Xu 2025 (35)	30/30	cirrhosis	Heidou sijun decoction +ETV/ETV; 12w	①③	√
Yin 2020 (36)	37/38	cirrhosis	Shugan Jianpi Formula +ETV/ETV; 6 m	①②③	√
Zhang 2021 (37)	50/49	cirrhosis	Jianpi Huoxue Lishui Fang+ETV/ETV; 4w	①③	√
Zhang 2023 (13)	47/76	liver fibrosis or cirrhosis	Xiaozhang decoction +ETV/ETV; 12 m	①②③④	NA
Zheng 2025 (38)	20/20	cirrhosis	Janpi Huoxue Shenshi Recipe+ETV/ETV; 3 m	①②③	√
Zhong 2017 (39)	44/44	cirrhosis	Yiqi Hexue formula +ETV/ETV; 24w	①	√
Zou 2020 (40)	33/33	cirrhosis	Yiqi Hexue formula +ETV/ETV; 4w	①	NA

T, treatment; C, control; ETV, entecavir; ADV, adefovir; TDF or TAF, tenofovir; AR, adverse reactions; NA, not applicable; Observation indicators: ① immune index (CD3+, CD4+, CD8+, CD4+/CD8+, NK, IL-6, IL-8, TNF-A, and TNF-B); ② liver fibrosis index (HA, LN, PC-III, IV-C) or LSM; ③ liver function (ALT, AST, TBIL, ALB); ④ the virological marker (HBV-DNA).

**Table 2:** Compositions (herbs) of TCM.

RCTs	Composition (herbs) of TCM
Chen 2019 (19)	Fuzheng Huayu capsule: Radix et Rhizoma Salviae Miltiorrhizae (Dan Shen), Gynostemma Pentaphyllum (Jiao Gu Lan), Pini Pollen (Song Hua Fen), Semen Persicae (Tao Ren), Fructus Schisandrae Chinensis (Wu Wei Zi)
Chen 2021 (20)	Zishen Rougan decoction: Trionycis Carapax (Bie Jia), Radix Rehmanniae (Di Huang), Angelicae Sinensis Radix (Dang Gui), Radix et Rhizoma Salviae Miltiorrhizae (Dan Shen), Moutan Cortex (Mu Dan Pi), Radix Curcumae (Yu Jin), Radix Glehniae (Bei Sha Shen), Lycii Fructus (Gou Qi Zi), Codonopsis Radix (Dang Shen), Ophiopogonis Radix (Mai Dong), Flos Mume (Lv E Mei), Coptidis Rhizoma (Huang Lian)
Duan 2021 (11)	Rougan Huaxian granules: Radix Astragali (Huang Qi), Coicis Semen (Yi Yi Ren), Rhizoma Polygonati (Huang Jing), Lycii Fructus (Gou Qi Zi), Fructus Jujubae (Da Zao), Ostreae Concha (Mu Li), Trionycis Carapax (Bie Jia), Herba Lycopi (Ze Lan), Galli Gigerii Endothelium Corneum (Ji Nei Jin), Exocarpium Citri Rubrum (Ju Hong), Polygoni Cuspidati Rhizoma et Radix (Hu Zhang), Moutan Cortex (Mu Dan Pi)
Feng 2022 (21)	Fufang Bie Jia Ruangan tablet: Trionycis Carapax (Bie Jia), Curcumae Rhizoma (E Zhu), Paeoniae Radix Rubra (Chi Shao), Angelicae Sinensis Radix (Dang Gui), Notoginseng Radix et Rhizoma (San Qi), Codonopsis Radix (Dang Shen), Radix Astragali (Huang Qi), Placenta Hominis (Zi He Che), Cordyceps (Dong Cong Xia Cao), Isatidis Radix (Ban Lan Gen), Fructus Forsythiae (Lian Qiao)
Gao 2023 (22)	Yigan Huashi decoction: Radix Pseudostellariae (Tai Zi Shen), Radix Astragali (Huang Qi), Radix et Rhizoma Salviae Miltiorrhizae (Dan Shen), Radix Paeoniae Alba (Bai Shao), Radix Bupleuri (Chai Hu), Trionycis Carapax (Bie Jia), Rhizoma Atractylodis Macrocephalae (Bai Zhu), Poria (Fu Ling), Polygoni Cuspidati Rhizoma et Radix (Hu Zhang), Herba Hedyotis Diffusae (Bai Hua She She Cao), Sedi Herba (Cui Pen Cao), Gynostemma Pentaphyllum (Jiao Gu Lan)
Gong 2025 (23)	Anluo Huaxian pill: Radix Rehmanniae (Di Huang), Notoginseng Radix et Rhizoma (San Qi), Hirudo (Shui Zhi), Bombyx Batryticatus (Jiang Can), Pheretima (Di Long), Rhizoma Atractylodis Macrocephalae (Bai Zhu), Radix Curcumae (Yu Jin), Calculus Bovis (Niu Huang), Concha Arcae (Wa Leng Zi), Moutan Cortex (Mu Dan Pi), Radix et Rhizoma Rhei (Da Huang), Hordei Fructus Germinatus (Mai Ya), Galli Gigerii Endothelium Corneum (Ji Nei Jin), Cornu Bubali (Shui Niu Jiao)
Guo 2025 (24)	Tongluo Ruangan decoction: Radix et Rhizoma Salviae Miltiorrhizae (Dan Shen), Codonopsis Radix (Dang Shen), Radix Astragali (Huang Qi), Scorpio (Quan Xie), Flos Carthami (Hong Hua), Bombyx Batryticatus (Jiang Can), Hirudo (Shui Zhi), Citri Reticulatae Pericarpium (Chen Pi)
Jiang 2021 (25)	Chaishao Liujun decoction: Radix Paeoniae Alba (Bai Shao), Radix et Rhizoma Ginseng (Ren Shen), Radix Bupleuri (Chai Hu), Rhizoma Atractylodis Macrocephalae (Bai Zhu), Poria (Fu Ling), Citri Reticulatae Pericarpium (Chen Pi), Pinelliae Rhizoma (Ban Xia), Radix et Rhizoma Glycyrrhizae (Gan Cao)
Li 2017 (26)	TCM: Cornu Bubali (Shui Niu Jiao), Radix Scrophulariae (Xuan Shen), Ophiopogonis Radix (Mai Dong), Coptidis Rhizoma (Huang Lian), Fructus Forsythiae (Lian Qiao), Radix Rehmanniae (Di Huang), Bambusa Emeiensis (Zhu Ye Xin), Radix et Rhizoma Salviae Miltiorrhizae (Dan Shen)
Li 2024 (27)	Xiangsha Liujunzi decoction: Radix et Rhizoma Salviae Miltiorrhizae (Dan Shen), Codonopsis Radix (Dang Shen), Rhizoma Atractylodis Macrocephalae (Bai Zhu), Radix Astragali (Huang Qi), Citri Reticulatae Pericarpium (Chen Pi), Fructus Amomi (Sha Ren), Poria (Fu Ling), Radix Aucklandiae (Mu Xiang), Pinelliae Rhizoma (Ban Xia), Semen Plantaginis (Che Qian Zi), Rhizoma Alismatis (Ze Xie), Radix Glycyrrhizae Praeparata (Zhi Gan Cao)
Lin 2025 (28)	Fuzheng Huayu capsule: the ame as Chen2019 (19).
Liu 2023 (29)	Ruangan Kangxian formula: Ostreae Concha (Mu Li), Paeoniae Radix Rubra (Chi Shao), Radix Astragali (Huang Qi), Poria (Fu Ling), Radix Sophorae Flavescentis (Ku Shen), Trionycis Carapax (Bie Jia), Radix et Rhizoma Salviae Miltiorrhizae (Dan Shen), Semen Persicae (Tao Ren), Curcumae Rhizoma (E Zhu), Fructus Crataegi (Shan Zha), Herba Lycopi (Ze Lan)
Ma 2021 (30)	Rougan Huazhuo Bushen formula: Angelicae Sinensis Radix (Dang Gui), Radix Paeoniae Alba (Bai Shao), Fructus Schisandrae Chinensis (Wu Wei Zi), Codonopsis Radix (Dang Shen), Rhizoma Atractylodis Macrocephalae (Bai Zhu), Poria (Fu Ling), Toosendan Fructus (Chuan Lian Zi), Rhizoma Corydalis (Yan Hu Suo), Radix Glycyrrhizae Praeparata (Zhi Gan Cao), Herba Duchesneae Indicae (She Mei), Radix et Rhizoma Salviae Miltiorrhizae (Dan Shen), Fructus Ligustri Lucidi (Nv Zhen Zi)
Niu 2023 (31)	Fuzheng Huayu capsule: the ame as Chen2019 (19).
Song 2021 (32)	Fuzheng Huayu capsule: the ame as Chen2019 (19).
Sun 2018 (12)	Yinzhi Ganfu granules: Artemisiae Scopariae Herba (Yin Chen), Gardeniae Fructus (Zhi Zi), Radix et Rhizoma Rhei (Da Huang), Herba Hedyotis Diffusae (Bai Hua She She Cao), Polyporus (Zhu Ling), Radix Bupleuri (Chai Hu), Angelicae Sinensis Radix (Dang Gui), Radix Astragali (Huang Qi), Codonopsis Radix (Dang Shen), Radix et Rhizoma Glycyrrhizae (Gan Cao)
Sun 2022 (33)	Fuzheng Qudu decoction: Radix Astragali (Huang Qi), Angelicae Sinensis Radix (Dang Gui), Herba Taraxaci (Pu Gong Yin), Salviae Chinensis Herba (Ban Zhi Lian), Herba Hedyotis Diffusae (Bai Hua She She Cao), Radix Bupleuri (Chai Hu), Radix Curcumae (Yu Jin), Radix Glycyrrhizae Praeparata (Zhi Gan Cao)
Xie 2025 (34)	Fuzheng Huayu capsule: the ame as Chen2019 (19).
Xu 2025 (35)	Heidou Sijun decoction: Sojae Semen Nigrum (Hei Dou), Codonopsis Radix (Dang Shen), Rhizoma Atractylodis Macrocephalae (Bai Zhu), Poria (Fu Ling), Cinnamomi Ramulus (Gui Zhi), Radix Paeoniae Alba (Bai Shao), Radix Glycyrrhizae Praeparata (Zhi Gan Cao), Angelicae Sinensis Radix (Dang Gui), Fructus Tribuli (Ci Ji Li), Plastrum Testudinis (Gui Ban), Trionycis Carapax (Bie Jia), Hordei Fructus Germinatus (Mai Ya)
Yin 2020 (36)	Shugan Jianpi formula: Radix Bupleuri (Chai Hu), Radix Astragali (Huang Qi) Rhizoma Atractylodis Macrocephalae (Bai Zhu), Poria (Fu Ling), Cortex Magnoliae Officinalis (Hou Po), Citri Reticulatae Pericarpium (Chen Pi), Radix Curcumae (Yu Jin), Fructus Aurantii (Zhi Qiao), Radix Paeoniae Alba (Bai Shao), Angelicae Sinensis Radix (Dang Gui), Radix et Rhizoma Salviae Miltiorrhizae (Dan Shen), Radix Glycyrrhizae Praeparata (Zhi Gan Cao)
Zhang 2021 (37)	Jianpi Huoxue Lishui decoction: Notoginseng Radix et Rhizoma (San Qi), Poria (Fu Ling), Rhizoma Alismatis (Ze Xie), Polyporus (Zhu Ling), Radix Paeoniae Alba (Bai Shao), Radix Astragali (Huang Qi), Pericarpium Arecae (Da Fu Pi), Semen Plantaginis (Che Qian Zi), Achyranthis Bidentatae Radix (Niu Xi)
Zhang 2023 (13)	Xiaozhang decoction: Radix Pseudostellariae (Tai Zi Shen), Rhizoma Atractylodis Macrocephalae (Bai Zhu), Citri Reticulatae Pericarpium (Chen Pi), Poria (Fu Ling), Herba Lycopi (Ze Lan), Rhizoma Alismatis (Ze Xie), Galli Gigerii Endothelium Corneum (Ji Nei Jin), Ostreae Concha (Mu Li), Trionycis Carapax (Bie Jia)
Zheng 2025 (38)	Janpi Huoxue Shenshi Recipe: Coicis Semen (Yi Yi Ren), Curcumae Rhizoma (E Zhu), Poria (Fu Ling), Campsis Flos (Ling Xiao Hua), Radix Astragali (Huang Qi), Fructus Polygoni Orientalis (Shui Hong Hua Zi), Radix Bupleuri (Chai Hu), Pheretima (Dilong), Fructus Amomi (Sha Ren)
Zhong 2017 (39)	Yiqi Hexue formula: Radix Astragali (Huang Qi), Angelicae Sinensis Radix (Dang Gui), Galli Gigerii Endothelium Corneum (Ji Nei Jin), Herba Lycopi (Ze Lan), Trionycis Carapax (Bie Jia)
Zou 2020 (40)	Yiqi Hexue formula: Radix Astragali (Huang Qi), Puerariae Lobatae Radix (Ge Gen), Paeoniae Radix Rubra (Chi Shao), Radix et Rhizoma Rhei (Da Huang), Radix et Rhizoma Salviae Miltiorrhizae (Dan Shen), Radix Curcumae (Yu Jin), Rhizoma Sparganii (San Leng), Curcumae Rhizoma (E Zhu), Flos Carthami (Hong Hua), Trichosanthis Fructus (Gua Lou)

### 3.3 Results of methodological quality evaluation

**Figure 3:**
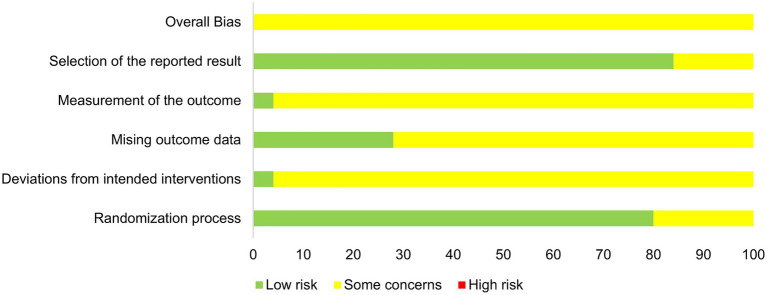
Risk of bias graph.

**Figure 4:**
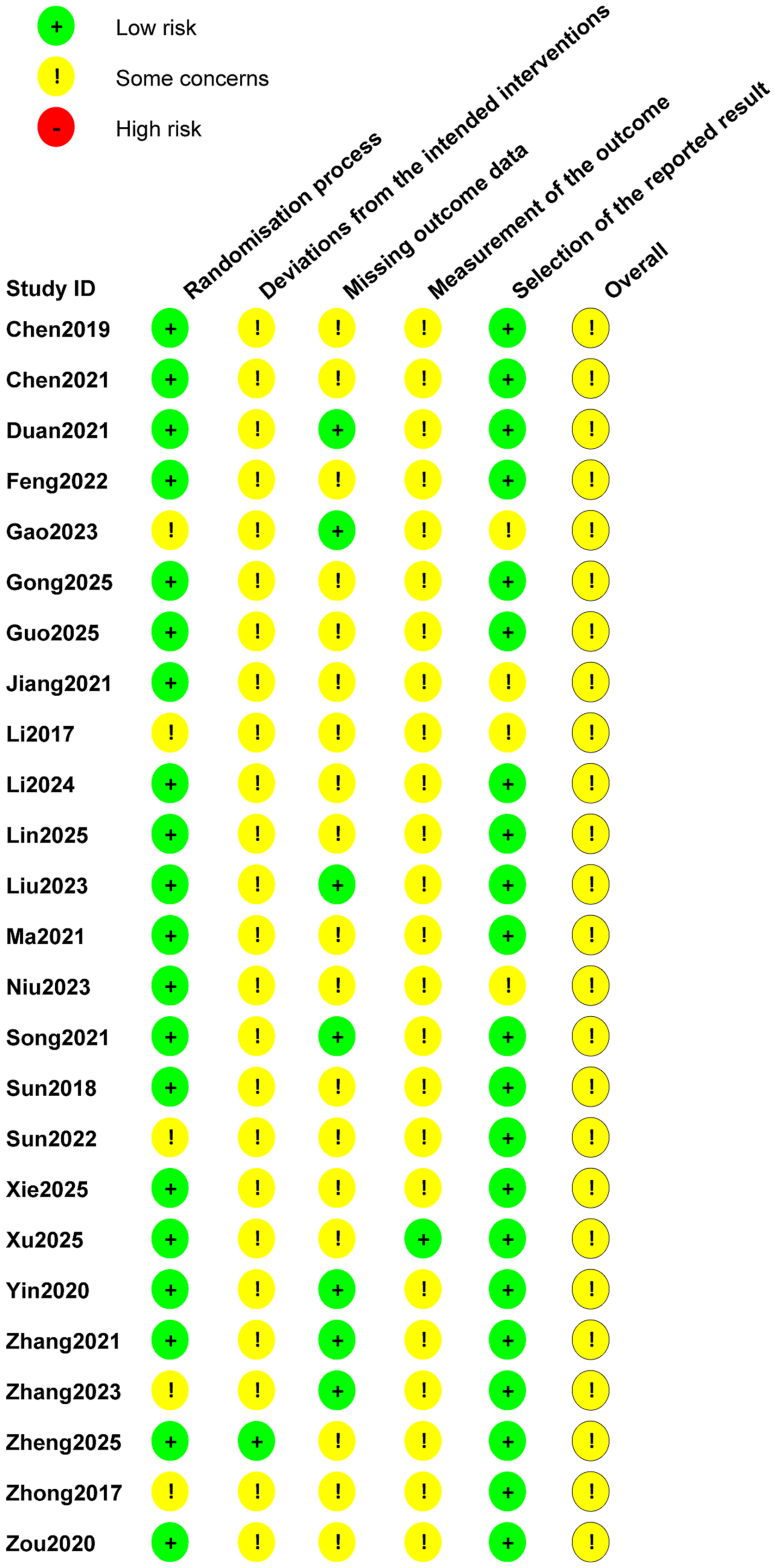
Risk of bias summary.

Among these trials, 5 trials (13, 22, 26, 33, 38) only mentioned random grouping but did not describe the specific grouping method, and the remaining studies mentioned specific random grouping. Additionally, one trial (35) specified the blinding method used for outcome indicator assessment, and another trial (38) mentioned the use of a TCM placebo. Seven trials (11, 13, 22, 29, 32, 36, 37) mentioned incomplete outcome data or analyzed cases lost to follow-up. Four trials (22, 25, 26, 31) lacked sufficient information to determine the presence of selective reporting of results, whereas the other trials did not exhibit selective reporting. Other methodological items without relevant clear descriptions were classified as “some concerns exist.” The risk of bias graph and summary are presented in Figures 3, 4.

### 3.4 Meta-analysis results of TCM combined with NAs in treating hepatitis B-related liver fibrosis/cirrhosis

#### 3.4.1 Meta-analysis of immune function

**Figure 5:**
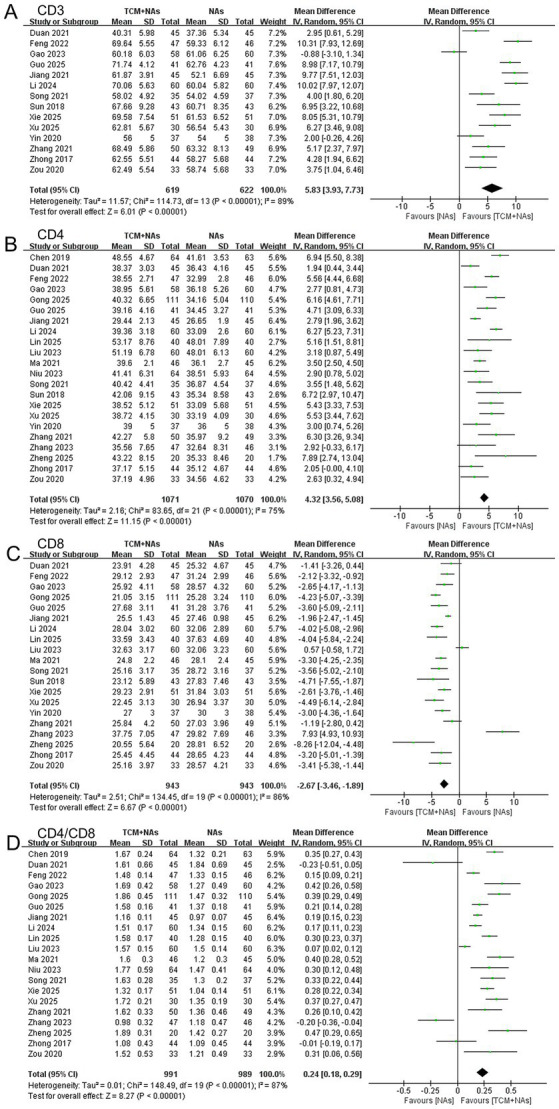
Meta-analysis of T lymphocyte subsets. **(A)** Meta-analysis of CD3+. **(B)** Meta-analysis of CD4+. **(C)** Meta-analysis of CD8+. **(D)** Meta-analysis of CD4+/CD8 + .

The primary indicators related to the immune function index, containing CD3+, CD4+, CD8+, CD4+/CD8+, NK cells, and cytokines, were assessed. Significant heterogeneity among immune cells was observed, with an I^2^ value exceeding 50%, indicating substantial variability. Consequently, random effects models were employed for the meta-analysis. The findings, depicted in the forest plot in Figure 5, suggest that TCM combined with NAs could increase levels of CD3 + (MD = 5.83; 0.95CI = 3.93, 7.73; *p* < 0.00001), CD4 + (MD = 4.23; 0.95CI = 3.56, 5.08; *p* < 0.00001), and the CD4+/CD8 + ratio (MD = 0.24; 0.95CI = 0.18, 0.29; *p* < 0.00001), while decreasing CD8 + levels (MD = -2.67; 0.95CI = -3.46, −1.89; *p* < 0.00001). Additionally, TCM may enhance NK cell activity and elevate certain cytokines, such as IL-6, TGF-*β*, and TNF-*α*, as detailed in Table 3.

**Table 3:** Meta-analysis of other immune indexes.

Indicators	Study	Sample (T/C)	I^2^/%	Model	MD	95%CI	*p*
NK	3 (13, 19, 30)	157/154	96	random	5.12	[1.50, 8.74]	0.006
IL-6	4 (20, 26, 32, 33)	242/244	95	random	−6.17	[−11.22, −1.11]	0.02
IL-8	2 (26, 33)	151/151	98	random	−5.19	[−15.03, 4.66]	0.30
TGF-β	2 (12, 20)	99/99	94	random	−52.87	[−94.49, −11.25]	0.01
TNF-α	4 (11, 26, 32, 33)	231/233	52	random	−3.68	[−6.27, −1.10]	0.005

#### 3.4.2 Meta-analysis of HA, LN, IV-C, PC-III, and LSM

**Figure 6:**
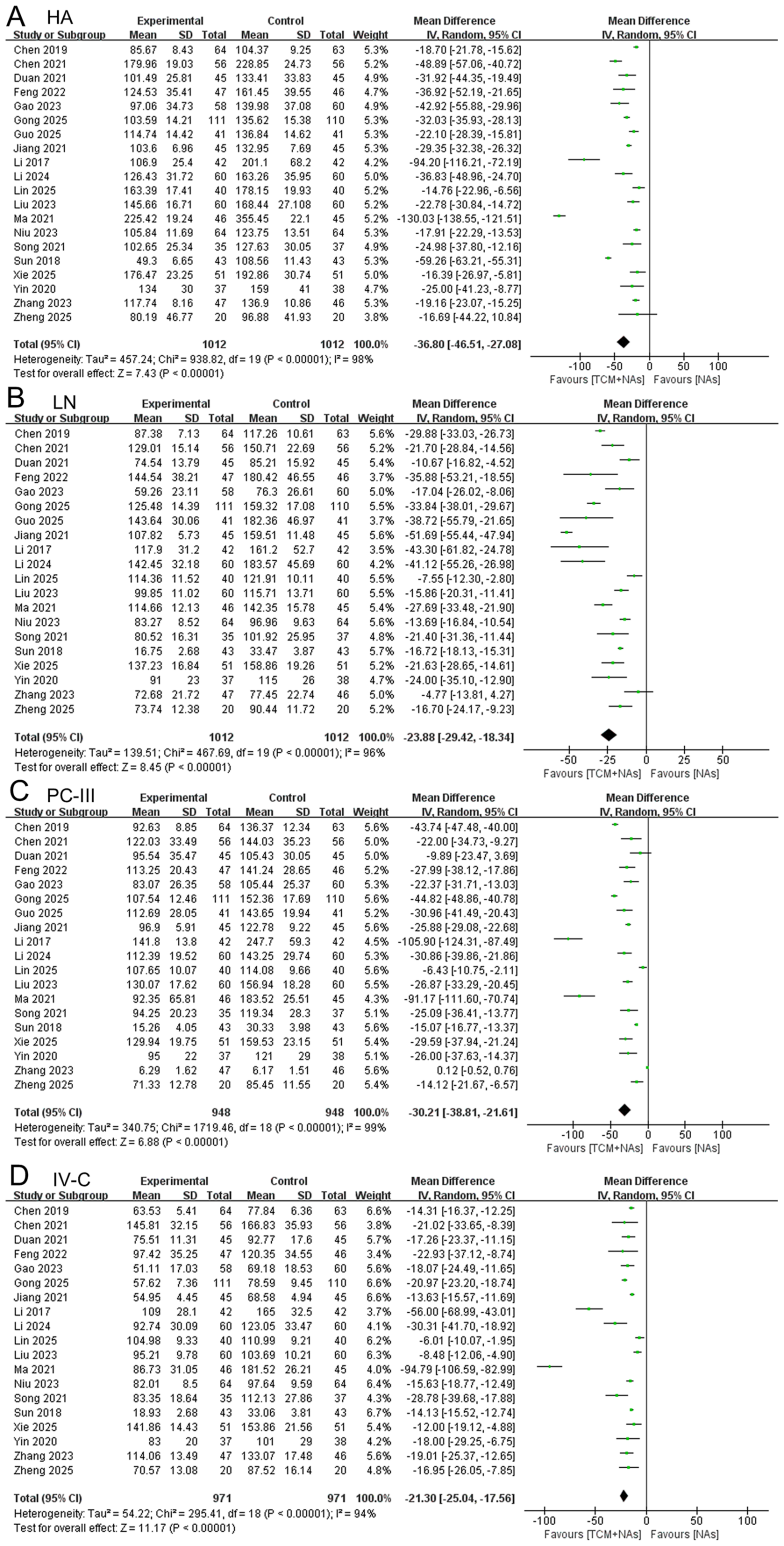
Meta-analysis of liver fibrosis indexes. **(A)** Meta-analysis of HA. **(B)** Meta-analysis of LN. **(C)** Meta-analysis of PC-III. **(D)** Meta-analysis of IV-C.

**Figure 7:**
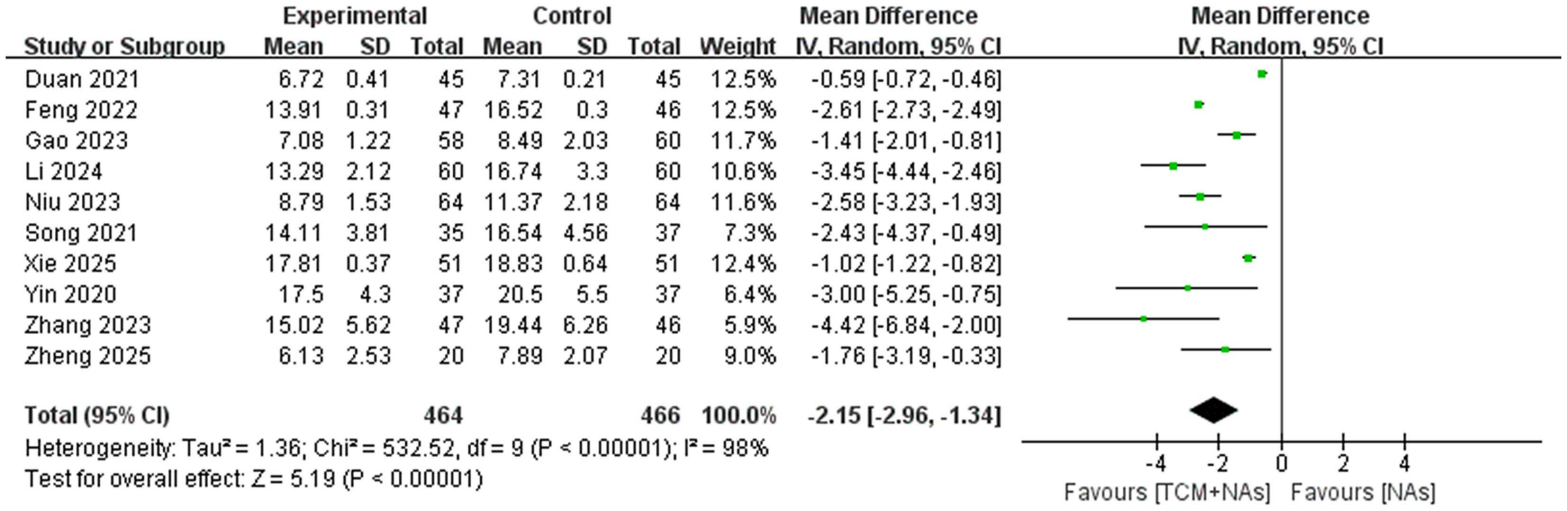
Meta-analysis of LSM.

Twenty articles reported the liver fibrosis indexes and LSM. The values of I^2^ of the heterogeneity test were greater than 50%, suggesting high heterogeneity, and the random effect model was utilized for meta-analysis. The results of meta-analysis demonstrated that TCM combined with NAs could decrease the HA, LN, PC-III, IV-C (Figure 6), and LSM (Figure 7), whose effects were superior to NAs alone for hepatitis B-related liver fibrosis/cirrhosis.

#### 3.4.3 Meta-analysis of improving liver function

**Figure 8:**
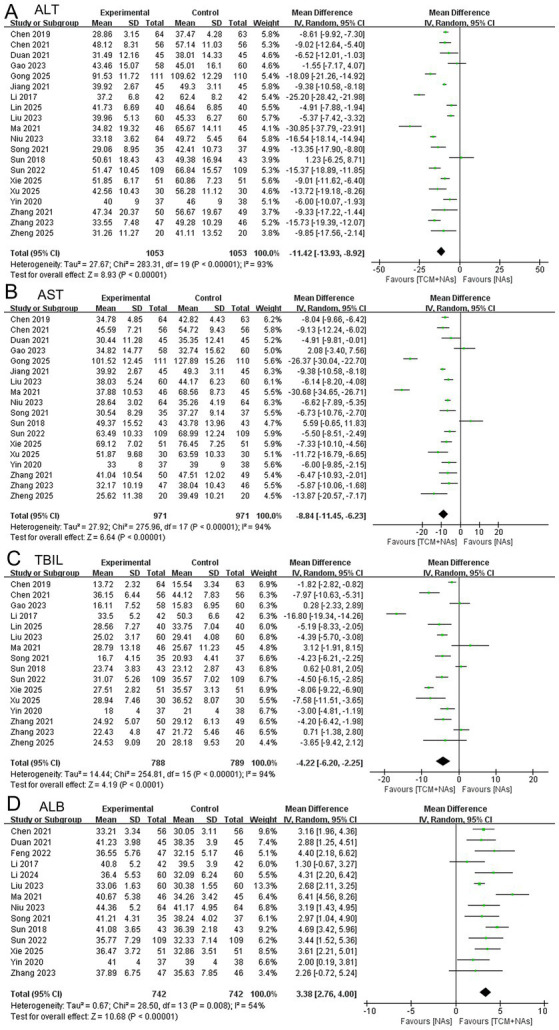
Meta-analysis of liver function. **(A)** Meta-analysis of ALT. **(B)** Meta-analysis of AST. **(C)** Meta-analysis of TBIL. **(D)** Meta-analysis of ALB.

Twenty-two articles mentioned liver function. The heterogeneity statistic I2 exceeded 50%, indicating high heterogeneity, and the random-effect model was used for meta-analysis. The results of meta-analysis showed that TCM combined with NAs could decrease the levels of ALT, AST, and TBIL, while increasing ALB levels (Figure 8).

#### 3.4.4 Analysis for other observation indicators and safety evaluation

Two trials (13, 20) demonstrated that TCM combined with NAs may contribute to decreased serum HBV-DNA loads. One RCT showed that TCM combined with NAs could increase the conversion rate of HBV-DNA, hepatitis B surface antigen (HBsAg), and Hepatitis B e antigen (HBeAg), compared with the control group. However, another RCT (11) suggested that TCM combined with NAs could not reduce the serum HBV-DNA loads.

In addition, 14 trials reported the adverse reactions of interventions. No severe adverse reactions were observed in patients who were treated with TCM, and mild adverse reactions such as gastrointestinal symptoms (nausea and vomiting) (20) were reported in individual patients. Seven trials (24, 27–29, 31, 35, 39) reported that there was no statistical difference in adverse reactions between the treatment groups and control groups. Six trials (11, 12, 26, 36–38) reported that treatment groups experienced no adverse reactions.

#### 3.4.5 Analyses for publication bias and quality of the evidence

**Figure 9:**
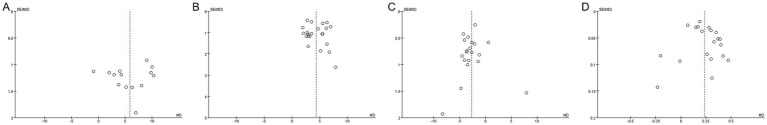
Funnel plot analysis for immune function. **(A)** Funnel plot of CD3 + cells. **(B)** Funnel plot of CD4 + cells. **(C)** Funnel plot of CD8+. **(D)** Funnel plot of CD4+/CD8 + .

In immune function, if no fewer than 10 trials were included, a publication bias analysis was conducted using funnel plots. As shown in Figure 9, it was determined that the distribution of the funnel plot was slightly asymmetrical. All outcomes were included in a GRADE evidence profile in Table 4. Except for ALB, the evidence on other indicators was of low quality.

**Table 4:** Grading evidence of the included literature.

Certainty assessment							Group		Statistical measure	Certainty
Outcomes	RCTs	Risk of bias	Inconsistency	Indirectness	Imprecision	Other considerations	TCM + NAs	NAs		
CD3	14	not serious	serious	not serious	not serious	strongly suspected	619	622	MD 5.83 higher (3.93 higher to 7.73 higher)	Low
CD4	22	not serious	serious	not serious	not serious	strongly suspected	1,071	1,070	MD 4.32 higher (3.56 higher to 5.08 higher)	Low
CD8	20	not serious	serious	not serious	not serious	strongly suspected	943	943	MD 2.67 lower (3.46 lower to 1.89 lower)	Low
CD4/CD8	20	not serious	serious	not serious	not serious	strongly suspected	991	989	MD 0.24 higher (0.18 higher to 0.29 higher)	Low
HA	20	not serious	serious	not serious	not serious	strongly suspected	1,012	1,012	MD 36.8 lower (46.51 lower to 27.08 lower)	Low
LN	20	not serious	serious	not serious	not serious	strongly suspected	1,012	1,012	MD 23.88 lower (29.42 lower to 18.34 lower)	Low
PC-III	19	not serious	serious	not serious	not serious	strongly suspected	948	948	MD 30.21 lower (38.81 lower to 21.61 lower)	Low
IV-C	19	not serious	serious	not serious	not serious	strongly suspected	971	971	MD 21.3 lower (25.04 lower to 17.56 lower)	Low
LSM	10	not serious	serious	not serious	not serious	strongly suspected	464	466	MD 2.15 lower (2.96 lower to 1.34 lower)	Low
ALT	20	not serious	serious	not serious	not serious	strongly suspected	1,053	1,053	MD 11.42 lower (13.93 lower to 8.92 lower)	Low
AST	18	not serious	serious^a^	not serious	not serious	strongly suspected	971	971	MD 8.84 lower (11.45 lower to 6.23 lower)	Low
TBIL	16	not serious	serious	not serious	not serious	strongly suspected	788	789	MD 4.22 lower (6.2 lower to 2.25 lower)	Low
ALB	14	not serious	not serious	not serious	not serious	strongly suspected	742	742	MD 3.38 higher (2.76 higher to 4 higher)	Moderate

## 4 Discussion

HBV, a hepatotropic virus, can cause liver cell damage, immune cell infiltration, and liver fibrosis, ultimately progressing to hepatitis B-related cirrhosis. The immune pathogenesis of hepatitis B-related liver fibrosis and cirrhosis involves intricate virus-host interactions, marked by dysregulated T-cell responses and cytokine production (8, 41). In contrast, non-viral cirrhosis is driven by sterile inflammation resulting from metabolic stress and damage-associated molecular patterns (8). The immune system plays a crucial role in the onset and progression of hepatitis B-related liver fibrosis and cirrhosis. By modulating immune cell activity, novel strategies for anti-fibrosis/cirrhosis therapies can be developed. Consequently, the investigation of therapeutic agents for hepatitis B-related liver fibrosis/cirrhosis has emerged as a significant area of research.

Complementary and alternative medicine is extensively utilized in the management of liver fibrosis and cirrhosis. TCM may offer therapeutic benefits in cirrhosis by modulating immune mechanisms, thereby inhibiting liver fibrosis, reducing oxidative stress, and suppressing inflammatory responses (42). Clinically, TCM demonstrates notable advantages in the treatment of cirrhosis. For instance, the combination of Bushen Huayu Decoction and entecavir has been shown to significantly improve liver function indicators and alleviate symptoms of liver fibrosis in patients with compensated cirrhosis (43). Furthermore, a seven-year open-label follow-up cohort study indicated that Biejia Ruangan could enhance the efficacy of entecavir in reversing hepatitis B-related liver cirrhosis (44). Nevertheless, there remains a paucity of systematic, evidence-based medical research regarding the role of TCM in modulating immune functions in patients with hepatitis B-related liver fibrosis/ cirrhosis.

In order to establish the evidence of TCM in regulating immune functions in patients with hepatitis B-related liver fibrosis/cirrhosis, 25 trials that met the inclusion criteria were analyzed with meta-analyses in the study. Risk of bias evaluations suggested some concerns for the majority of RCTs. The result of meta-analysis showed that compared with NAs alone, TCM combined with NAs can increase a variety of immune cells (CD3+, CD4+, CD4+/CD8+, NK), and reduce cytokines (IL-6, TGF-*β*, TNF-*α*) and CD8 + cells in patients with hepatitis B-related liver fibrosis/cirrhosis. The meta-analysis of immune function revealed substantial heterogeneity, potentially attributable to variations in baseline values among the included studies or the course of treatment. Notably, the pooled MDs for CD3+, CD4+, CD8, and the CD4+/CD8 + ratio remained stable, and the statistical significance of these pooled MDs was unaffected by the exclusion of any single study.

The activation of HSCs is a key step in the development of liver fibrosis and cirrhosis, and immune cells such as T cells and NK cells regulate the activation of HSCs and the fibrosis process by secreting cytokines and chemokines (45). Chronic HBV infection disrupts T-cell immunity, contributing to the progression of liver fibrosis and cirrhosis. Following infection, CD4^+^ T lymphocytes are more likely to undergo apoptosis, reducing their proportion while CD8^+^ cells increase, thereby lowering the CD4^+^/CD8^+^ ratio—a well-recognized indicator of immune imbalance (46, 47). CD4 + T cells play a crucial role in the immune response during HBV infection. Among the conventional subsets of CD4 + T cells, T helper 1 (Th1) and Th2 cells are particularly notable. Th1 cells secrete high levels of IFN-*γ*, which can kill HSCs (48), and facilitate the development of a robust, specific antiviral immune response and mitigate tissue fibrosis (49). Analysis of single-cell transcriptome data and fluorescence-activated cell sorting has revealed a progressive decline in the NK cell population, whereas CD8 + T cells are expanded in fibrotic liver tissue (50). This observation underscores the protective role of NK cells (51) and confirms that CD8 + T cells infiltrate fibrotic liver tissue, thereby contributing to the progression of fibrosis (52). Besides, the CD3 molecule is a critical component of the T-cell receptor complex, participating in T-cell activation and signal transduction. Previous research found that the ratio of circulating CD3 + T cells was decreased in chronic hepatitis B and HBV-related acute-on-chronic liver failure patients compared with healthy controls (53). However, no study has yet investigated the relationship between CD3 + T cells and hepatitis B-related liver fibrosis/cirrhosis in depth. In terms of cytokines, levels of TGF-*β* and TNF-*α* are increased in individuals with advanced fibrosis. Additionally, HSCs can mount a proinflammatory response upon stimulation with TNF-α or TGF-β1 (54). Furthermore, enhanced TGF-*β* signaling further promotes the activation of HSCs and the progression of liver fibrosis (55). In the presence of inflammation, IL-6 can activate the Janus kinase 2 (JAK2)-signal transducer and activator of transcription 3 (STAT3) signaling pathway, leading to liver fibrosis (56). Thus, HBV may contribute to the progression of liver fibrosis/cirrhosis through the disruption of immune homeostasis, characterized by a decreased CD4^+^/CD8^+^ T cell ratio (impaired anti-fibrotic activity), an expansion of pro-fibrotic CD8^+^ T cells, and a reduction in NK cells; the infection elevates levels of pro-fibrotic cytokines, including TGF-β, TNF-*α*, and IL-6, which potentially activate HSCs. In this meta-analysis, these peripheral immune changes suggest systemic immune modulation rather than direct tissue-level mechanisms. While alterations in lymphocyte subsets and cytokines support the idea of systemic immunomodulation by TCM, they do not prove similar immune events occur in target tissues, as tissue-level analyses were not conducted in the RCTs. Future research with tissue-specific immune studies is needed to confirm a direct mechanistic link.

The meta-analysis of the liver fibrosis index and liver function indicates that the combination of TCM with NAs is significantly more effective than NAs alone. Several studies also reported that the integration of TCM with NAs may lead to a reduction in serum HBV-DNA levels. This suggests that TCM, when combined with NAs, may inhibit HBV replication, enhance liver function, and ameliorate liver fibrosis by modulating immune cell activity, thereby contributing to the treatment of liver fibrosis or cirrhosis. Therefore, TCM may exert anti-HBV-related liver fibrosis/cirrhosis effects by restoring immune balance, improving the CD3 and CD4^+^/CD8^+^ ratio, reducing profibrotic CD8^+^ T cells, rescuing protective NK cells, and inhibiting profibrotic cytokines. Notably, heterogeneity was detected among the included studies, potentially attributable to variations in treatment duration or baseline index values. Despite the relatively high degree of heterogeneity, we conducted sensitivity analyses excluding studies one by one, and found the results of immune function, liver fibrosis index, and liver function were stable. Furthermore, among the 14 trials that reported adverse events, no serious reactions were observed, and the addition of TCM was not associated with an increase in adverse events. Regarding the primary outcome measures, the funnel plot analysis suggests the presence of potential small-study or publication bias. Primary outcomes in this review were rated as low certainty by the GRADE framework, and the pooled results should be approached with caution, acknowledging that the true effect might differ significantly from the reported estimates. These findings are preliminary and not a solid basis for treatment recommendations.

Although there were important discoveries revealed by this study, some limitations in this meta-analysis still exist. Firstly, substantial between-study heterogeneity was observed. TCM interventions are generally guided by pattern differentiation, which can be individualized or applied to a group sharing the same pattern. Strict inclusion criteria would not reflect the real-world practice of TCM and would have excluded a large proportion of the available evidence. The included studies used a wide variety of herbal compositions, with almost every study applying a different modified TCM formula. Grouping them into a small number of compositionally similar categories would still leave each subgroup with fewer studies, precluding valid statistical analysis. Reporting of some variables (e.g., treatment duration, disease severity) was inconsistent or incomplete across trials. This prevented us from constructing reliable covariates for meta-regression or meaningful subgroups. Moreover, 11 out of the 25 included trials (44%) did not provide any safety data. The absence or incomplete documentation of safety does not imply the absence of adverse events, and firm conclusions about TCM’s clinical safety cannot be made based on the current evidence. In addition, various versions of Chinese guidelines for diagnosing liver fibrosis/cirrhosis were employed in the inclusion criteria. While the diagnostic criteria of these guidelines are basically the same, some diagnostic conditions may be slightly different. Different versions of guidelines do not alter the final diagnosis but may influence the severity assessment of patients’ conditions, impacting the summary results and their heterogeneity. The heterogeneity may limit the direct clinical translation of our findings. Last but not least, the evidence base is weakened by several methodological flaws. Many trials poorly reported their randomization process, often lacking details on sequence generation, raising concerns about selection bias. Allocation concealment was frequently unreported or poorly executed, further increasing this risk. Blinding, especially for outcome assessors, was often undocumented, leading to potential detection bias. Incomplete outcome data and selective reporting were hard to assess due to the lack of published prospective protocols. Thus, the overall quality of included studies is relatively low. Although the trend toward improvement in immune function is encouraging, the results of meta-analysis need to be interpreted cautiously.

In the context of analyzing the compositions (herbs) of TCM, Poria cocos, Rhizoma Atractylodis Macrocephalae (Bai Zhu), Poria (Fu Ling), and Radix Paeoniae Alba (Bai Shao) demonstrate relatively high levels of support and confidence, frequently co-occurring. According to TCM theory, this combination is believed to fortify the spleen and soften the liver. Additionally, Radix et Rhizoma Salviae Miltiorrhizae (Dan Shen) and Semen Persicae (Tao Ren) are commonly combined with Gynostemma Pentaphyllum (Jiao Gu Lan), Pini Pollen (Song Hua Fen), and Fructus Schisandrae Chinensis (Wu Wei Zi) to establish a compatibility model aimed at reinforcing deficiency and alleviating blood stasis. These herbs are also primary constituents of the Fuzheng Huayu capsule. Fuzheng Huayu capsule is commonly used in treating liver fibrosis and cirrhosis in China, and research shows Fuzheng Huayu capsule’s anti-fibrotic effects include inhibiting hepatic stellate cell activation, reducing inflammation, protecting liver cells, and promoting liver regeneration (9). The potential mechanisms of Fuzheng Huayu capsule in treating HBV-fibrosis are not clear, and our findings offer some immune-related clues. Furthermore, the combination of Codonopsis Radix (Dang Shen), Angelicae Sinensis Radix (Dang Gui), and Radix Astragali (Huang Qi) exhibits a support value of 0.20, representing the foundational model for nourishing both Qi and blood. The polysaccharide of Rhizoma Atractylodis Macrocephalae (Bai Zhu) could significantly promote T cell activation in animal models (57, 58). Polysaccharide of Radix et Rhizoma Salviae Miltiorrhizae (Dan Shen) specifically promotes the proliferation and enhances cytotoxicity of T lymphocytes in peripheral blood of cancer patients through activation of toll-like receptors (TLRs) mediated-mitogen activated protein kinase (MAPK) and - nuclear factor kappa-B (NF-κB) pathways (59). Rats with chronic fatigue syndrome that received Angelicae Sinensis Radix (Dang Gui) and Radix Astragali (Huang Qi) exhibited corrected T cell subsets counts and decreased mRNA levels of IL-1β, TNF-*α* (60). These herbs exhibit multiple mechanisms and significant potential in regulating immune cells’ function, revealing the potential application value of TCM in modern medicine and providing new ideas for the development of new drugs. Nevertheless, it should be noted that some of the mechanistic inferences discussed herein are derived from non-hepatitis disease models, and their direct relevance to HBV-related liver fibrosis/cirrhosis requires further validation in appropriate experimental and clinical settings.

To sum up, meta-analysis results suggested that TCM with NAs could improve immune function, liver fibrosis, and liver function of hepatitis B-related liver fibrosis or cirrhosis patients. Given the existence of high heterogeneity and asymmetrical funnel plots showing potential bias, caution is warranted in interpreting these results. Due to the limitations of sample size, literature quality, and publication bias, more high-quality and large-sample RCTs are needed to support our results, and future prospective studies focusing on specific, standardized TCM interventions are warranted.

## Data Availability

The original contributions presented in the study are included in the article/Supplementary material, further inquiries can be directed to the corresponding author.

## Footnotes

1
https://www.yiankb.com/

2
https://www.gradepro.org/
