# Supplementary material for: The efficacy of traditional Chinese medicine on immune function in patients with hepatitis B-related liver fibrosis or cirrhosis: a systematic review and meta-analysis
Source: Front Med (Lausanne). 2026 Jul 20;13:1872781. doi: 10.3389/fmed.2026.1872781 (PMC13429670; doi:10.3389/fmed.2026.1872781)
Supplement: Supplementary file 1 [file Data_Sheet_1.docx]

Supplementary Material

# Supplementary Data

**Search terms used in each database**

**Search terms used in PubMed**

(((((("Hepatitis B"[Mesh] OR "Hepatitis B, Chronic"[Mesh]) OR (HBV[Title/Abstract])) OR (hepatitis B[Title/Abstract])) AND (((((("Liver Cirrhosis"[Mesh]) OR (cirrhosis[Title/Abstract])) OR (cirrhotic[Title/Abstract])) OR (hepatocirrhosis[Title/Abstract])) OR (hepatic fibrosis[Title/Abstract])) OR (liver fibrosis[Title/Abstract]))) AND ((("Medicine, Chinese Traditional"[Mesh]) OR (Chinese medicine[Title/Abstract])) OR (Chinese materia medica[Title/Abstract]))) AND (("Immunity, Cellular"[Mesh]) OR (immune[Title/Abstract]))) AND (("Randomized Controlled Trial" [Publication Type] OR "Randomized Controlled Trials as Topic"[Mesh]) OR (randomized[Title/Abstract]))

**Search terms used in Embase**

('hbv'/exp OR 'hbv' OR 'hepatitis b'/exp OR 'hepatitis b') AND ('cirrhosis'/exp OR cirrhosis OR cirrhotic OR hepatocirrhosis OR 'hepatic fibrosis'/exp OR 'hepatic fibrosis' OR 'liver fibrosis'/exp OR 'liver fibrosis') AND ('chinese medicine'/exp OR 'chinese medicine' OR 'chinese materia medica'/exp OR 'chinese materia medica') AND ('immune'/exp OR immune) AND randomized

**Search terms used in Web of Science**

|  | **Search** |
| --- | --- |
|  | #5 AND #4 AND #3 AND #2 AND #1 |
| #5 | TS=(randomized) |
| #4 | TS=(immune) |
| #3 | (TS=(Chinese medicine)) OR TS=(Chinese materia medica) |
| #2 | ((((TS=(cirrhosis)) OR TS=(cirrhotic)) OR TS=(hepatocirrhosis)) OR TS=(hepatic fibrosis)) OR TS=(liverfibrosis) |
| #1 | (TS=(HBV)) OR TS=(hepatitis b) |

**Search terms used in Cochrane database**

|  | **Search** |
| --- | --- |
| #1 | (HBV):ti,ab,kw OR (hepatitis b):ti,ab,kw |
| #2 | MeSH descriptor: [Hepatitis B] this term only |
| #3 | MeSH descriptor: [Hepatitis B, Chronic] this term only |
| #4 | #1 OR #2 OR #3 |
| #5 | (cirrhotic).ti,ab,kw OR (cirhosis):ti,ab,kw OR (hepatocirrhosis).ti,ab,kw OR (hepatic fibrosis).ti,ab,kw OR (iver fibrosis)ti,ab,kw |
| #6 | MeSH descriptor: [Liver Cirrhosis] this term only |
| #7 | #5 OR #6 |
| #8 | (Chinese medicine):ti,ab,kw OR (Chinese materia medica):ti,ab,kw |
| #9 | MeSH descriptor: [Medicine, Chinese Traditional] this term only |
| #10 | #8 OR #9 |
| #11 | (immune):ti,ab,kw |
| #12 | MeSH descriptor: [Immunity, Cellular] this term only |
| #13 | #11 OR #12 |
| #14 | (Randomized):ti,ab,kw |
| #15 | MeSH descriptor: [Randomized Controlled Trial] this term only |
| #16 | #14 OR #15 |
| #17 | #4 AND #7 AND #10 AND #13 AND #16 |

**Search terms used in CNKI**

|  | **Search** |
| --- | --- |
| #1 | Topics: 乙型肝炎 + 乙肝 + 乙型病毒肝炎 + 乙型病毒性肝炎 + HBV + hepatitis b |
| #2 | Topics: 肝硬化 + 肝硬变 + 肝纤维化 + 肝脏纤维化 |
| #3 | Topics: 中医 + 中医药 + 中药 |
| #4 | Topics: 免疫 |
| #5 | Topics: 随机 |
|  | #1 AND #2 AND #3 AND #4 AND #5 |

**Search terms used in Wanfang**

|  | **Search** |
| --- | --- |
| #1 | Topics: 乙型肝炎 OR 乙肝 OR 乙型病毒肝炎 OR 乙型病毒性肝炎 OR HBV OR hepatitis b |
| #2 | Topics: 肝硬化 OR 肝硬变 OR 肝纤维化 OR 肝脏纤维化 |
| #3 | Topics: 中医 OR 中医药 OR 中药 |
| #4 | Topics: 免疫 |
| #5 | Topics: 随机 |
|  | #1 AND #2 AND #3 AND #4 AND #5 |

**Search terms used in CBM**

|  | **Search** |
| --- | --- |
| #1 | Common fields: 乙型肝炎 OR 乙肝 OR 乙型病毒肝炎 OR 乙型病毒性肝炎 OR HBV OR hepatitis b |
| #2 | Common fields: 肝硬化 OR 肝硬变 OR 肝纤维化 OR 肝脏纤维化 |
| #3 | Common fields: 中医 OR 中医药 OR 中药 |
| #4 | Common fields: 免疫 |
| #5 | Common fields: 随机 |
|  | #1 AND #2 AND #3 AND #4 AND #5 |

**Search terms used in Weipu**

|  | **Search** |
| --- | --- |
| #1 | Abstract: 乙型肝炎+乙肝+乙型病毒肝炎+乙型病毒性肝炎+HBV+hepatitis b |
| #2 | Abstract: 肝硬化+肝硬变+肝纤维化+肝脏纤维化 |
| #3 | Abstract: 中医 OR 中医药 OR 中药 |
| #4 | Abstract: 免疫 |
| #5 | Abstract: 随机 |
|  | #1 AND #2 AND #3 AND #4 AND #5 |
